# Paper-based colorimetric hyperammonemia sensing by controlled oxidation of plasmonic silver nanoparticles†

**DOI:** 10.1039/d4na00021h

**Published:** 2024-04-15

**Authors:** Padryk Merkl, Georgios A. Sotiriou

**Affiliations:** a Department of Microbiology, Tumor and Cell Biology, Karolinska Institutet SE-17177 Stockholm Sweden georgios.sotiriou@ki.se

## Abstract

High concentrations of ammonia in the human body can occur due to a wide variety of underlying causes such as liver cirrhosis and the symptoms of high ammonia concentrations are diffuse and hard to diagnose. The measurement of blood ammonia levels is an important diagnostic tool but is challenging to perform at the patient's bedside. Here, we present a plasmonic Ag nanoparticle-based ammonia sensor which provides a colorimetric optical readout and does not require specialised equipment. This is achieved using plasmonic Ag/SiO_2_ nanoparticles with the sensing mechanism that in the presence of OCl^−^ they rapidly degrade reducing their plasmonic extinction and losing their characteristic colour. However, if ammonia is also present in the system, it neutralises the OCl^−^ and thus the silver nanoparticles retain their plasmonic colour as can be measured by the naked eye or using a spectrometer. This sensing was further developed to enable measurements with animal serum as well as a implementing a facile “dip-stick” style paper-based sensor.

## Abstract 2

High ammonia concentrations in the human body are diffuse and hard to diagnose but can lead to serious neurological complications. To tackle this, we developed an ammonia sensor based on silver nanoparticles and demonstrated it in a dip-stick format.

## Graphical abstract

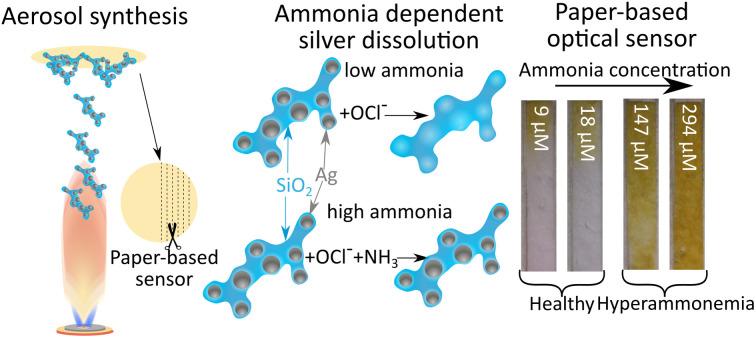

## 1 Introduction

Ammonia is a bioanalyte with strong links to metabolic health. Ammonia production largely occurs in the intestine where bacterial urease activity produces ammonia which can diffuse into the blood stream.^1^ The normal metabolic function of the human body also produces ammonia as a waste product. Ammonia can exist in both its ionised form (ammonium ion, NH_4_^+^), predominately found at physiological pH, and in the non-ionic NH_3_ form (here the term ammonia will be used to refer to the total content of both NH_3_ and NH_4_^+^).^2^ Ammonia can travel across cell membranes and the blood–brain barrier by diffusion and membrane transport proteins.^3^ A consequence of this is that elevated ammonia levels can directly affect the brain and lead to significant neurotoxicity and irreversible brain damage.^4^ The removal of ammonia by the liver is an important regulation pathway for the control of ammonia levels in the body. In adult humans hyperammonaemia is often defined as plasma ammonia levels > 50 μM.

The diffuse nature of hyperammonaemia, manifesting as symptoms ranging from minor cognitive or behavioural changes to coma, renders an easy and accurate assessment of circulating ammonia levels in the body essential. Today, blood ammonia levels are most often quantified by enzymatic assays in well-equipped hospital laboratories; however, the necessary instruments and specialised training of the personnel required to run the assays render their application in resource-limited settings challenging.^5^ Moreover, ammonia levels in blood samples can change rapidly after sampling, necessitating either a rigorous cold chain or immediate analysis.^6^ There is therefore great interest in the development of alternative methods for clinical ammonia analysis. A simple system for measuring ammonia levels in biological samples was recently developed using fluorescent dyes encapsulated in ammonia selective polymerosomes which is currently in the process of commercialisation through the company GENFIT.^7^ Commercially available point-of-care devices exist such as the PocketChem™ Blood Ammonia Analyzer (Arkray, Japan), which alkalises the ammonia thereby driving it into the gas phase and causing a colour change in a bromocresol green indicator strip.^8^ This system uses disposable strips and is suitable for bedside testing. This approach of applying gas phase sensing for direct ammonia determination was also recently applied using an ammonia fuel cell as the sensing element, with measurements within minutes.^9^ However, both methods rely on custom instruments to quantify ammonia levels which could limit their applicability in resource-limited settings.

One of the earliest methods to determine ammonia levels colorimetrically is the Berthelot reaction, first described in 1859, in which ammonia is reacted with the OCl^−^ ion to form a chloramine which can subsequently react with phenol to yield indophenol with a strong blue colour in alkaline environments.^10^ This method has since been optimised to reduce interference due to ionic strength; however, it is not suitable for the direct detection of ammonia from whole blood due to the interference of other amines present. Moreover, it requires potentially toxic reagents and specialised measurement equipment.^11^ Perfluorosulfonic acid polymers such as Nafion are often used as ion exchange membranes for the transport of small positively charged species and Nafion is extensively used in the fields of energy storage and electronics for its high capacity for proton exchange.^12^ Nafion was recently demonstrated for the effective separation of ammonia from whole blood by Ayyub *et al.*. This method allows ammonia to be collected in a clean and controlled buffer for subsequent analysis by the Berthelot reaction.^13^ A custom optical detection module was subsequently designed by Pasqualotto *et al.* for readout of the Berthelot reaction from such a Nafion separation system.^14^ However, the reliance on the Berthelot reaction with indophenol and sodium nitroprusside again limits the applicability of this system in point-of-care settings. For point-of-care testing, inexpensive single-use colorimetric sensors are highly attractive, a requirement which the rapid developments in paper-based sensors aim to address.^15^

Plasmonic materials are attractive for such colorimetric sensors due to their strong coupling to visible light which can provide high sensitivity.^16,17^ Plasmonic Ag nanoparticles have previously been exploited as sensors for ammonia in liquids by detecting shifts in their plasmonic absorbance spectra due to changes in their local environment (*e.g.* adsorption or aggregation).^18,19^ However, this has been demonstrated only in pure water and at ammonia concentrations above the clinically relevant blood concentrations. Here, the aim was to develop an ammonia sensor using plasmonic Ag nanoparticles with high sensitivity in the clinically relevant range and robust detection in ionic buffers. The OCl^−^ ion can dissolve metallic silver as well as react with ammonia.^20^ This has been applied here as the sensing mechanism, whereby a high ammonia concentration neutralises and decreases the OCl^−^ concentration resulting in limited dissolution of the Ag nanoparticles. In contrast, a low ammonia concentration leaves a high OCl^−^ concentration and therefore dissolution of the Ag occurs that removes its plasmonic colour. The ion transport properties of Nafion membranes were applied for the selective separation of ammonia from serum mimics and sheep serum. To further improve the ease of use and potential for a simple and inexpensive point-of-care application, a paper-based “dip-stick” ammonia sensor was developed that can distinguish relevant ammonia concentrations for hyperammonaemia.

## 2 Results and discussion

### 2.1 Ammonia sensing using plasmonic silver in solution

**Fig. 1:**
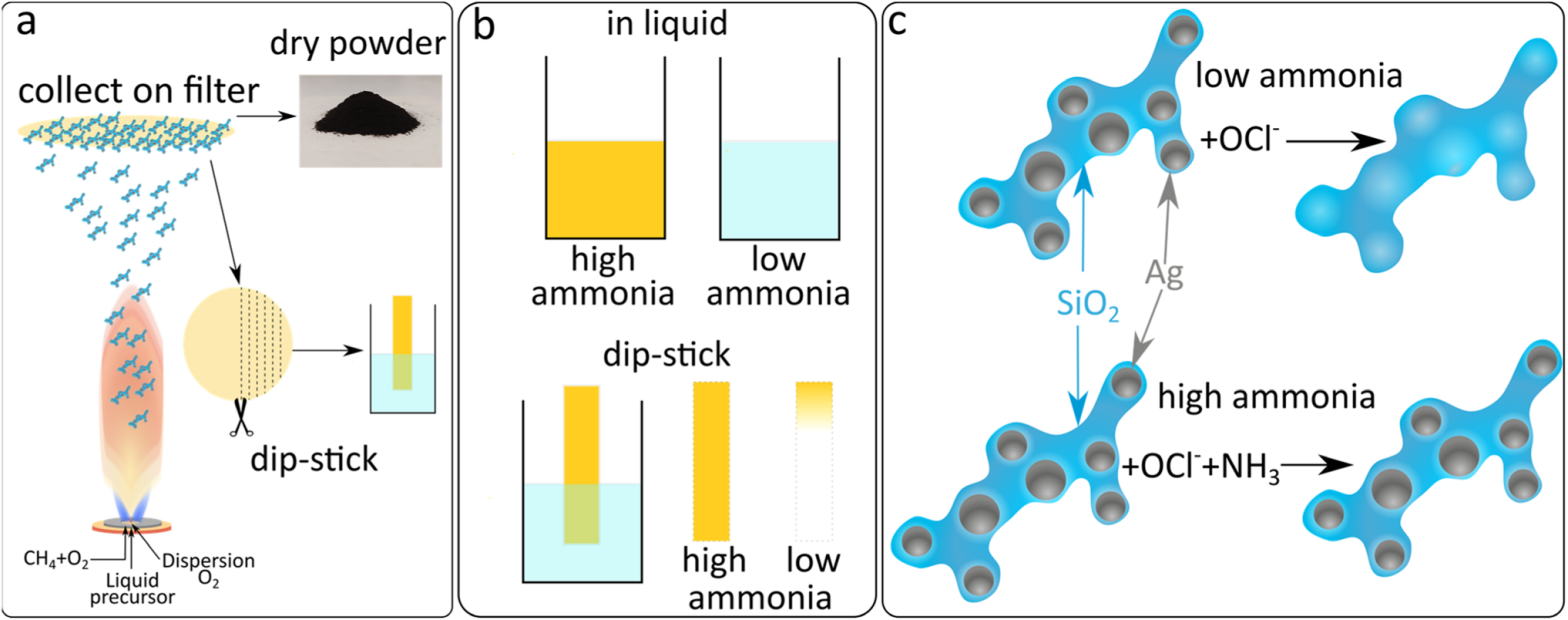
(a) Schematic description of the synthesis of Ag nanoparticles and collection on a glass fibre filter paper with the aid of a vacuum pump. Subsequently the Ag nanoparticles can be scraped off the filter as a dry powder ready for dispersion in the chosen medium or the filter can be cut into thin strips which can be directly used in a dip-stick format. (b) the two sensing modes applied, liquid phase dispersion of the nanoparticles and paper-based dip-stick style sensing. In both cases ammonia dependent removal of the silver nanoparticles leads to a colour change which provides the readout. (c) Schematic depiction of the reaction leading to the silica supported silver nanoparticle-based ammonia sensitivity. OCl^−^ ions can oxidatively dissolve silver nanoparticles to their Ag(i) ionic form or to form AgCl. However, if ammonia is present it will react with the OCl^−^ ions and thereby prevent the dissolution of the silver nanoparticles.

**Fig. 2:**
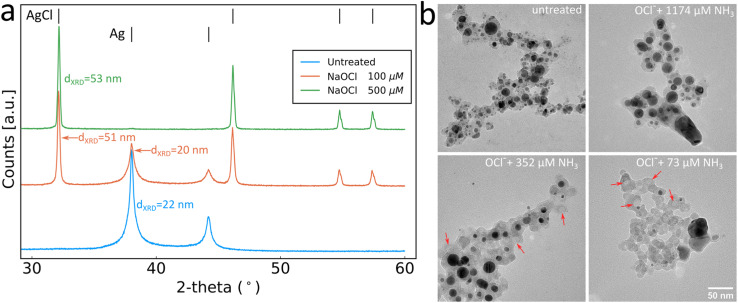
(a) XRD diffractograms of SiO_2_-coated Ag nanoparticles in the untreated state or with addition of different concentrations of NaOCl. (b) TEM images of SiO_2_-coated Ag nanoparticles untreated or treated with NaOCl in the presence of different concentrations of ammonia as indicated in the image. The scale bar in the lower right image applies to all images.

The production of sensing SiO_2_-coated Ag nanoparticles was performed by flame spray pyrolysis. The aerosol synthesised particles were subsequently collected by vacuum filtration and retrieved as a dry powder or directly used as collected on glass-fibre filters, as illustrated in Fig. 1a. This allows for the synthesis of particles to be used as a liquid phase sensor or as a paper-based sensor (Fig. 1b). The SiO_2_ coating of the Ag nanoparticles is essential to form well-separated Ag nanoparticles with desirable plasmonic properties by flame spray pyrolysis.^21–23^ The SiO_2_-content included influences the colloidal stability and primary particle size of Ag nanoparticles but does not interfere with the sensing mechanism. The sensing mechanism of this study relies on the dissolution of the Ag nanoparticles by OCl^−^ (schematically illustrated in Fig. 1c). To verify this behaviour, Ag nanoparticle suspensions were prepared in ultrapure water and exposed to 0, 100 and 500 μM NaOCl for 10 min. The suspensions were washed 3 times with ultrapure water by centrifugation and freeze-dried and XRD diffractograms were collected for the retrieved powders as shown in Fig. 2a. The diffractogram of the ultrapure water treated sample shows the characteristic peaks of pure Ag nanoparticles with average crystallite size *d*_XRD_ = 22 nm. At 100 μM NaOCl concentrations, AgCl peaks are observed with less intense Ag peaks and at 500 μM NaOCl the Ag peaks are almost completely attenuated and large AgCl peaks dominate with average crystallite size *d*_XRD_ = 53 nm. It should be noted that Ag^+^ ions are likely also released; however, these will be removed during washing steps.^20,24,25^ The effect of ammonia on the dissolution of the Ag nanoparticles was assessed by collecting TEM images of SiO_2_-coated Ag nanoparticles after exposure to 264 μM NaOCl and varying ammonia concentrations. As can be seen from Fig. 2b with the addition of sufficiently high ammonia concentrations the Ag nanoparticle structure could be almost entirely preserved. However, with gradually decreasing ammonia concentrations the darker Ag nanoparticles disappear and lighter patches are observed in the SiO_2_ coating (illustrated by the overlayed red arrows in Fig. 2b). The size distributions of the Ag nanoparticles also shift to larger sizes with decreasing ammonia concentrations as shown in ESI, Fig. S1.† This could be explained by the reaction of the oxidising OCl^−^ ion with the ammonia, thereby reducing the effective OCl^−^ concentration available for reaction with the Ag nanoparticles.

**Fig. 3:**
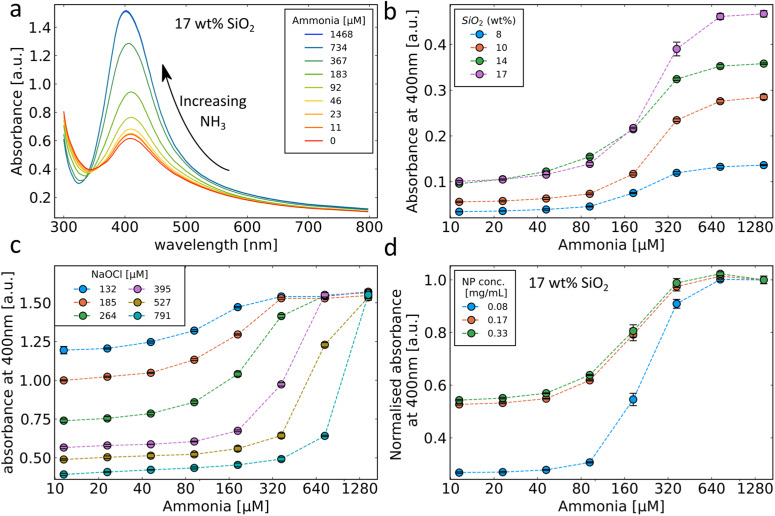
(a) Absorbance spectrum of SiO_2_-coated Ag nanoparticles (17 wt% SiO_2_) with addition of NaOCl in the presence of different concentrations of ammonia. (b) Performance of different silica wt% coatings in the presence of NaOCl and varying ammonia concentrations. (c) Performance of the 17 wt% silica coating in 4 different NaOCl concentrations and varying concentrations of ammonia. (d) Different concentrations of the 17 wt% silica coating in the presence of NaOCl and different ammonia concentrations. Ammonia concentrations in (b)–(d) are plotted on a log base 10 scale.

The strong plasmonic optical properties of Ag nanoparticles are affected by the nanoparticle dissolution that can be monitored by optical spectroscopy. To assess these changes in the optical properties of the Ag nanoparticles, varying concentrations of ammonia are prepared in PBS with 264 μM NaOCl. The Ag nanoparticle suspension is then added to this mixture to a final concentration of 0.125 mg mL^−1^ and the extinction spectra are recorded. The dissolution of the Ag nanoparticles by OCl^−^ and the protective effect of ammonia are clearly reflected in the plasmonic optical properties of the SiO_2_-coated Ag nanoparticles as can be observed in Fig. 3a. The typical plasmonic Ag absorbance peak at 400 nm is observed at high ammonia concentrations (and therefore low OCl^−^ concentrations). As the ammonia concentration decreases the plasmonic Ag is dissolved and therefore there is a decrease in the plasmonic absorbance peak at 400 nm. Previously, plasmonic Ag was used to directly sense ammonia by aggregation-induced plasmon shifts which can be sensitive to changes in ionic concentrations and was only performed in pure water.^18,19^ Here, we demonstrate successful ammonia sensing in PBS at clinically relevant concentrations (<50 μM). Nanoparticles often agglomerate in PBS due to high ionic strength; however, the presence of the SiO_2_ coating around the Ag nanoparticles here enables their dispersion and stability in PBS.^26–28^Fig. 3b demonstrates the ammonia sensing of Ag nanoparticles with different thicknesses of SiO_2_ coatings (varying SiO_2_ wt%). As the SiO_2_ wt% increases, the absorbance at high ammonia concentrations also increases. This is likely due to the increased stability of the higher SiO_2_ wt% coatings and the influence of the SiO_2_ wt% on Ag particle size rather than a direct effect of SiO_2_ in the sensing mechanism.^26^ The maximum difference between absorbance values at high and low ammonia concentrations is observed with 17 wt% SiO_2_ and is therefore used in further experiments.

The influence of different OCl^−^ concentrations on the ammonia sensing behaviour of the SiO_2_-coated Ag nanoparticles was evaluated and is shown in Fig. 3c. Since the OCl^−^ reacts with ammonia, the starting concentration of OCl^−^ has a large effect on the sensing range of the sensor. At high OCl^−^ concentrations, the absorbance decrease at low ammonia levels is high corresponding to a more complete removal of the Ag nanoparticles. The ammonia concentrations required to prevent a change in the absorbance also increase with increasing OCl^−^ concentrations. The influence of SiO_2_-coated Ag nanoparticle concentration on the sensor response is shown in Fig. 3d. At higher nanoparticle concentrations, the change in absorbance between high and low ammonia concentrations is smaller due to less removal of the Ag nanoparticles. Since the change in absorbance observed with increasing ammonia concentrations follows a sigmoidal curve, the absorbance values can vary dramatically over small changes in the ammonia concentration. Thus, this demonstrated ability to tune the location of the inflection point of the sensor response by modifying the concentrations of OCl^−^ nanoparticles can be important to achieve high sensitivity in the desired range.

### 2.2 Ammonia sensing with Nafion membranes

**Fig. 4:**
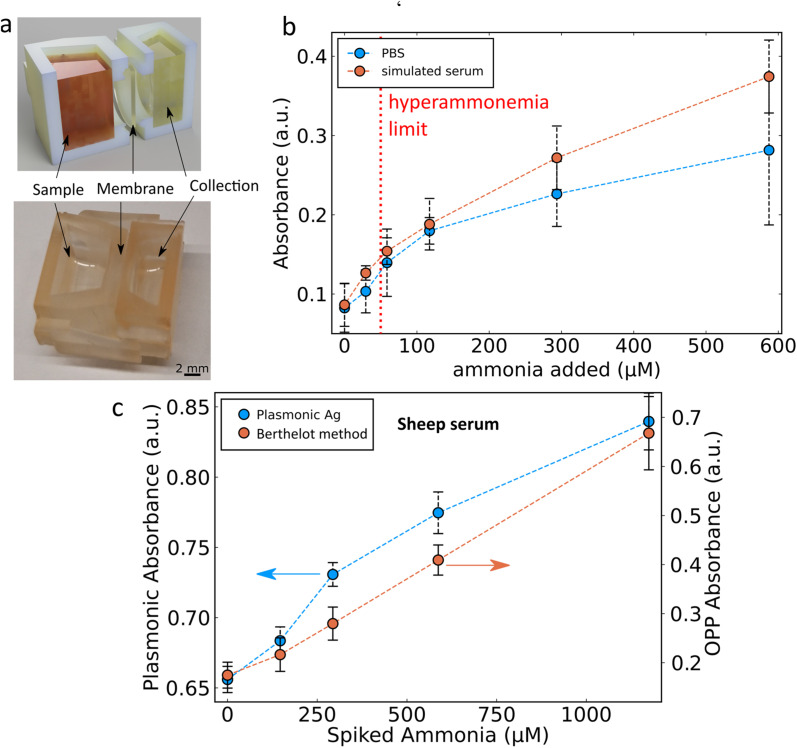
(a) Rendering (left) and digital image (right) of the Nafion-based ammonia separation system. (b) Separation of ammonia over 20 minutes from PBS (blue line) and simulated serum containing 50 mg per mL bovine serum albumin (orange line) with a red dotted line showing the blood ammonia limit for hyperammonaemia. (c) Separation of ammonia from spiked sheep's serum, showing the plasmonic silver ammonia sensor response (blue line) and the established Berthelot reaction (orange line).

A significant challenge with the proposed sensing mechanism is the potential for OCl^−^ to react with oxidisable molecules other than ammonia or SiO_2_-coated Ag nanoparticles. In the presence of similar protein concentrations to those found in plasma (50 mg mL^−1^ of bovine serum albumin) there is no sensitivity to ammonia as the OCl^−^ also reacts with the protein.^29^ Therefore, this would pose a serious challenge to the use of the SiO_2_-coated Ag nanoparticle ammonia sensor in human blood or plasma similarly to the challenge faced for the Berthelot reaction in such environments. Perfluorosulfonic acid polymers such as Nafion can be used in order to overcome this limitation by selectively separating ammonia from whole blood.^13^ A custom 3D printed chamber system was designed here where a high concentration of sodium acetate (3 M, pH 7.4) was placed on one side and a sample with high protein content was placed on the other side separated by a Nafion membrane as shown in Fig. 4a. This allowed for the separation and detection of ammonia from both PBS (Fig. 4b) and PBS with 50 mg per mL bovine serum albumin. The disadvantage of adding a Nafion membrane in the sensing system is the additional time required to allow for the transfer of ammonia across the membrane. Here, 20 min was chosen as a good compromise between speed and recovery (ESI, Fig. S2†) and for easier comparison with the literature.^13^ With the developed Ag nanoparticle-based system, it is possible to detect ammonia levels in such simulated serum and identify clinically high ammonia levels (red dotted line in Fig. 4b). The system was also evaluated with ammonia spiked samples of sheep serum as shown in Fig. 4c. Unspiked samples of sheep serum were found to initially have high levels of ammonia (>200 μM) due to protein degradation in storage in addition to the baseline levels ordinarily present in sheep.^6^ To reduce these levels of ammonia present in the serum Nafion membranes were used repeatedly with fresh sodium acetate buffer to separate the ammonia from the serum. However, this method was not able to completely remove the ammonia from the sheep serum and therefore a background ammonia level remained. Under these conditions the sensor was able to detect spiked ammonia with a limit of detection of 110 μM, non-linearity of 9.1% and sensitivity of 0.00015 a.u. per μM ammonia. To better understand the performance of the newly developed Ag nanoparticle-based sensor the well-established Berthelot method was used in parallel. The data shown in Fig. 4c demonstrate the ability of the nanoparticle-based ammonia sensor to perform as well as the Berthelot method in this challenging scenario. The selectivity of the developed plasmonic sensor in biological fluids is largely due to the Nafion membrane which has been extensively characterized for its high selectivity for such small positively charged ions as ammonia.^13,14,30^ Importantly, the nanoparticle-based ammonia sensor is more relevant in a resource-limited point-of-care setting as it can be adapted to a dip-stick style paper-based sensing system.

### 2.3 Ammonia sensing using paper-based systems

**Fig. 5:**
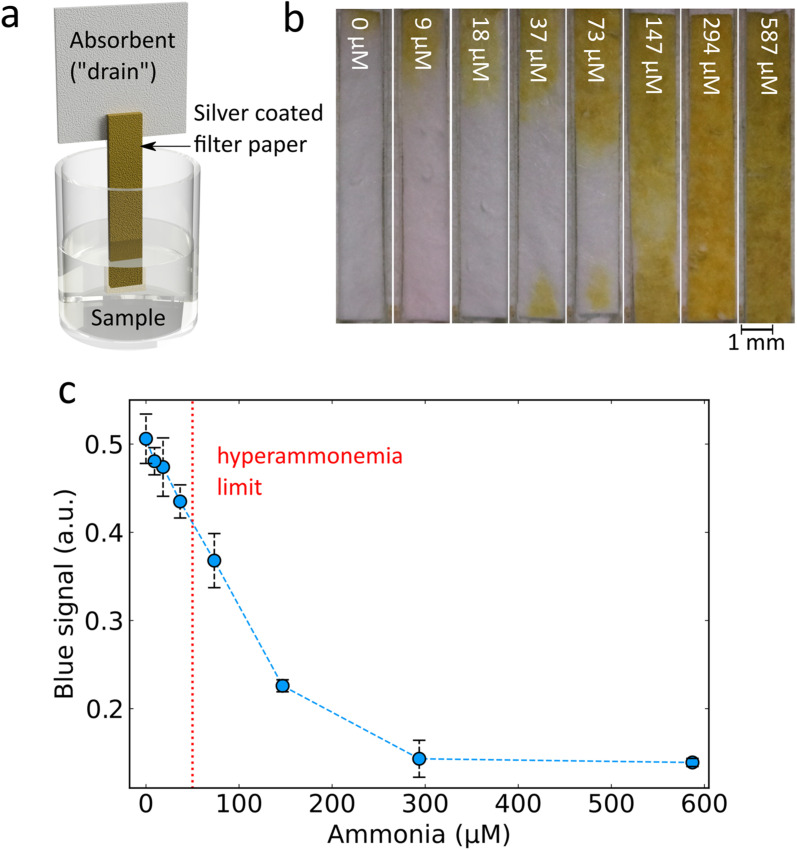
(a) Diagram of dip-stick style paper-based ammonia detection. (b) Digital photographs of paper-based ammonia sensors exposed to NaOCl, and different concentrations of ammonia as indicated in white writing overlayed on the digital images. (c) Measured blue channel signal by analysing digital photographs of the dip-stick style ammonia sensor, with a red dotted line showing the blood ammonia limit for hyperammonaemia. All silver deposited glass fibre filter paper shown here was synthesised with a 5 mL min^−1^ precursor flow rate dispersed using 5 L min^−1^ of oxygen with an 8 wt% SiO_2_ support with a deposition time of 5 s.

An important consideration for point-of-care applications is the simplicity of the assay required. Paper-based devices offer several advantages as they are cheap to fabricate, minimise liquid handling steps and are easy to dispose of.^15^ To achieve such a paper-based ammonia sensor, Ag nanoparticles synthesised in the aerosol phase were directly deposited with very short collection times (2–10 s) onto 257 mm diameter glass fibre filters. Flame-made Ag nanoparticles supported on amorphous SiO_2_ were chosen here as the more facile synthetic procedure which allowed for a more homogeneous coating of the filter paper.^31^ The flame synthesis conditions and silica wt% of the support were optimised to give the highest change between 300 μM ammonia and 0 μM ammonia (ESI, Fig. S3†) and therefore Ag deposited with a 5 mL min^−1^ precursor flow rate dispersed using 5 L min^−1^ of oxygen with an 8 wt% SiO_2_ support were chosen (full characterisation by XRD and N_2_ adsorption measurements of surface area for these different Ag nanoparticles can be found in ESI, Fig. S4 and Table S1,† respectively). Thin (1.2 mm × 10 mm) strips of Ag/SiO_2_ deposited filter paper were cut and combined with a cellulose backing to form a “dip-stick” style sensor (see Fig. 5a for a schematic). It should be noted that with our lab-scale flame aerosol reactor and deposition of nanoparticles on 257 mm diameter filter paper, it is possible to fabricate more than 2000 sensing strips/batch in a single step and within just a few seconds, highlighting the high scalability potential of this nanomanufacturing process.^32^

The ammonia aqueous samples were then prepared by adding increasing ammonia concentrations and a fixed concentration of OCl^−^. The Ag/SiO_2_ coated side of the dip-stick was then inserted. The liquid travels up through the paper by wicking and the addition of the cellulose backing provides an additional drain and allows enough liquid to flow through the sensitive Ag/SiO_2_ coated filter. Imaging of the resultant colour from the paper strips for increasing ammonia concentrations (Fig. 5b) reveals a clear visual trend consistent with the results obtained from solutions: an increased ammonia concentration preserves the strong plasmonic Ag colour while lower ammonia concentrations lead to higher removal of plasmonic Ag (ESI, Fig. S5†). By digital image analysis, it is possible to perform rapid quantification of the colour change (see ESI, Materials & methods† for details) revealing a sensitive response in the desired ppm range of around 50 μM (Fig. 5c). The sensing parameters of this paper-based system considering the range 0 to 147 μM are: limit of detection 47 μM, non-linearity 1.48% and a sensitivity of −0.0019 a.u. per μM ammonia. These results demonstrate that the developed dip-stick paper-based sensor can be used for determination of clinically relevant ammonia levels (red dotted line in Fig. 5c). With the use of a dip-stick paper strip instead of the liquid nanoparticle dispersion, the need for specialised equipment required for a nanoparticle dispersion and spectroscopy is eliminated, allowing more feasible translation of the sensor to point-of-care in resource-limited settings. The paper-based format allows the final sensor to be shipped dry which improves storage stability and decreases transit costs. The dip-stick application eliminates liquid transfer steps thereby improving ease of use and eliminating sources of user error. In addition, the measurement output is in a format which can easily be detected by the naked eye or using a phone camera and an appropriate mobile application. As such this paper-based sensor is preferable in point-of-care analysis.^33^ However, it should be noted that other substrate-based methods such as hydrogels can also provide sensitive point of use readouts.^34^

The developed paper-based sensor therefore shows potential as a point-of-care system according to the ASSURED criteria of the WHO.^35^ It is affordable (>2000 sensing strips from a single synthesis) and sufficiently sensitive to distinguish hyperammonemia. The Nafion membrane allows for specific detection. The paper-based format with colorimetric readout allows for user-friendly dip-stick style analysis without specialized equipment in under 30 minutes. Moreover, this dry format improves deliverability to the end user. However, it should be noted that further work must be carried out to confirm the viability of this sensor as a point-of-care system, such as a head-to-head comparison with existing commercial measurement technologies on clinical blood samples and a thorough analysis of sensor robustness and stability.

## 3 Conclusions

This work demonstrated the synthesis and optimisation of a novel ammonia sensor based on the reaction of OCl^−^ with both ammonia and plasmonic silver nanoparticles. The sensor was developed as both a colloidal dispersion compatible for well-plate format use and as a facile dip-stick style sensor that avoids the need for nanoparticle dispersions and allows read-out by the naked-eye or using a digital camera (*e.g.* a cell-phone camera). The sensing range of the sensor could be controlled by selecting the appropriate concentration of OCl^−^ and silver nanoparticles and was successfully developed to hit the biologically relevant window for hyperammonaemia. By exploiting the ammonia-selective properties of Nafion, the sensor was able to detect ammonia levels directly from serum in 20 min. This work therefore demonstrates the development of an easy to use and low-cost ammonia sensor which does not require specialised equipment for point-of-care applications in low resource settings.

## Data availability

Data supporting the findings of this study are available from the corresponding author upon request.

## Author contributions

Padryk Merkl: conceptualization; data curation; data collection; formal analysis; methodology; visualization; writing – original draft; writing – editing. Georgios A. Sotiriou: conceptualization; supervision; formal analysis; funding acquisition; methodology; resources; visualization; writing – original draft; writing – editing.

## Conflicts of interest

The authors declare no competing interests.

## Supplementary Material

NA-006-D4NA00021H-s001
