# Supplementary material for: Paper-based colorimetric hyperammonemia sensing by controlled oxidation of plasmonic silver nanoparticles
Source: Nanoscale Adv. 2024 Apr 15;6(10):2586–93. doi: 10.1039/d4na00021h (PMC11093257; doi:10.1039/d4na00021h)
Supplement: NA-006-D4NA00021H-s001 [file NA-006-D4NA00021H-s001.pdf]

Electronic Supplementary Material (ESI) for Nanoscale Advances.
This journal is © The Royal Society of Chemistry 2024

SUPPORTING INFORMATION

Paper-based colorimetric hyperammonemia sensing by controlled oxidation of plasmonic silver nanoparticles

*Padryk Merkl and Georgios A. Sotiriou**

Department of Microbiology, Tumor and Cell Biology, Karolinska Institutet, SE-17177 Stockholm, Sweden.

*Corresponding author: georgios.sotiriou@ki.se

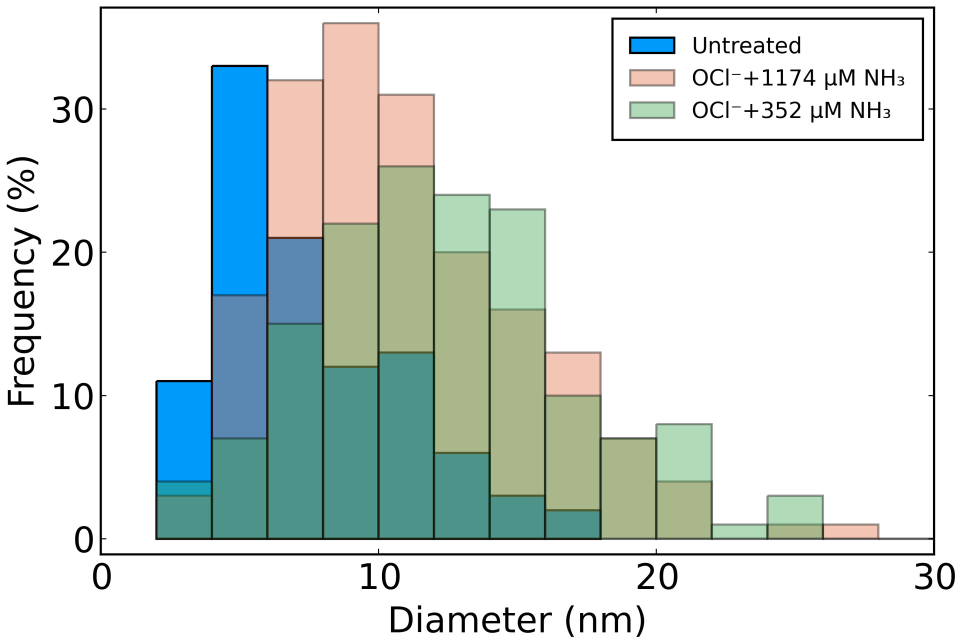

Figure S1: Particle size distributions assessed from TEM images of untreated (as-synthesised) particles and particles treated with OCl^- together with either high or low ammonia.

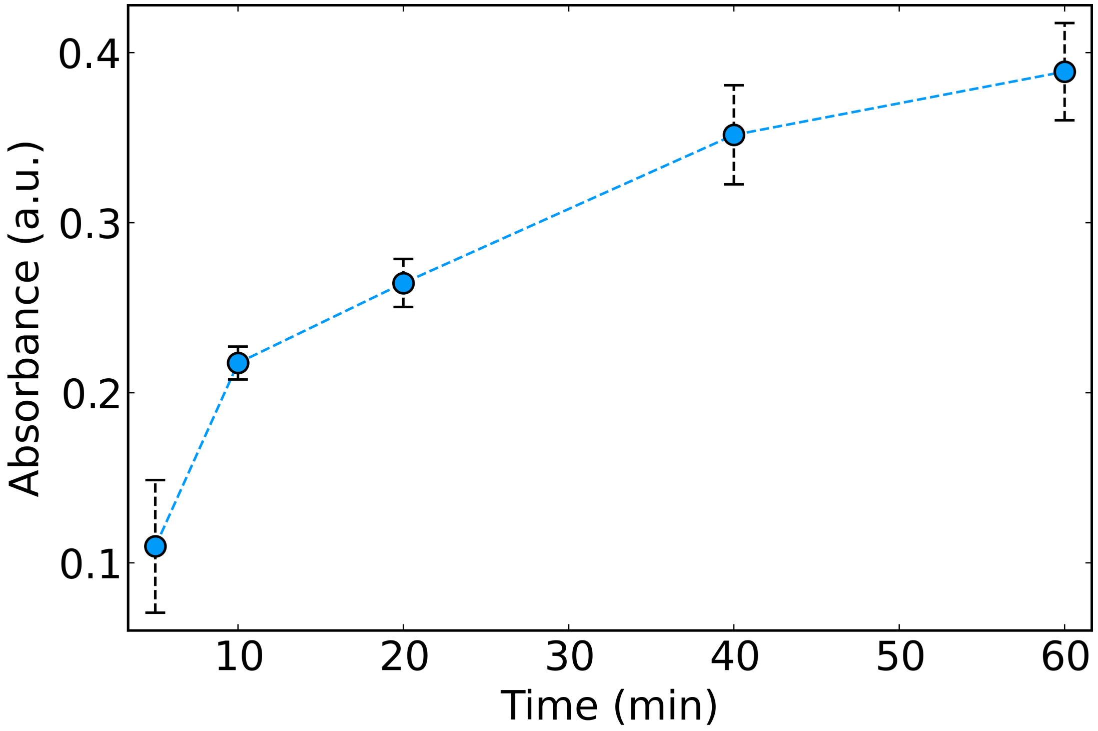

Figure S2: Kinetics of the ammonia transport across the nafion membrane from PBS

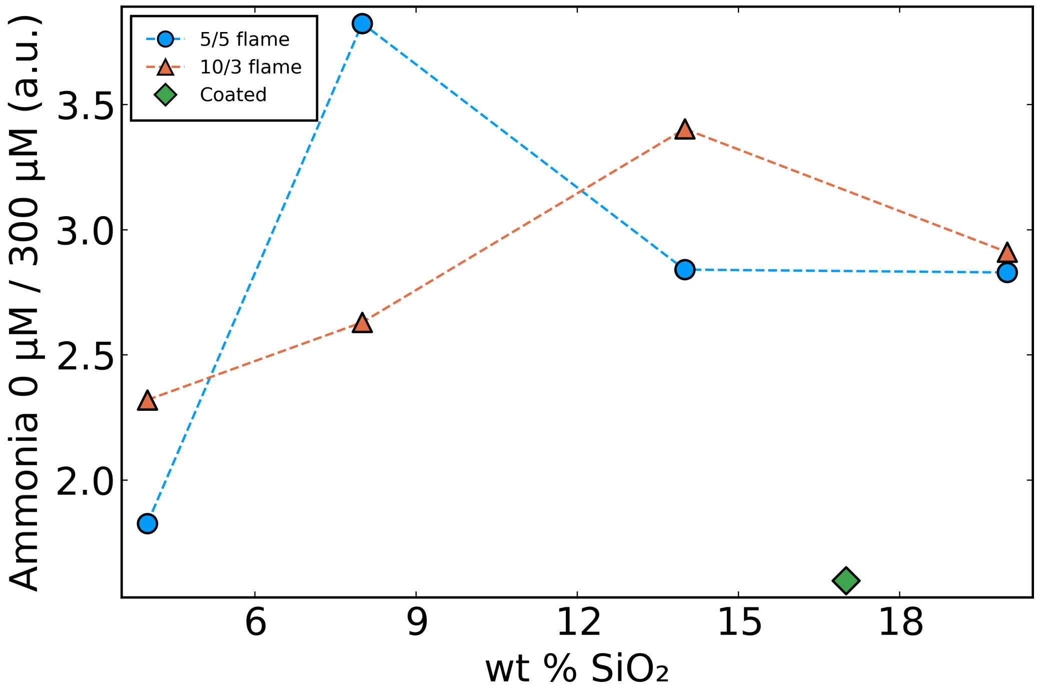

Figure S3: Evaluation of optimal silica wt% content as a support for silver nanoparticles deposited on glass fibre filters, defined as the ratio between the intensity of blue light without ammonia present and with 300 μM ammonia present. The terms X/X in the figure legend refer to the flame parameters of (liquid precursor flow rate [ml/min])/(dispersion oxygen flow rate [L/min]).

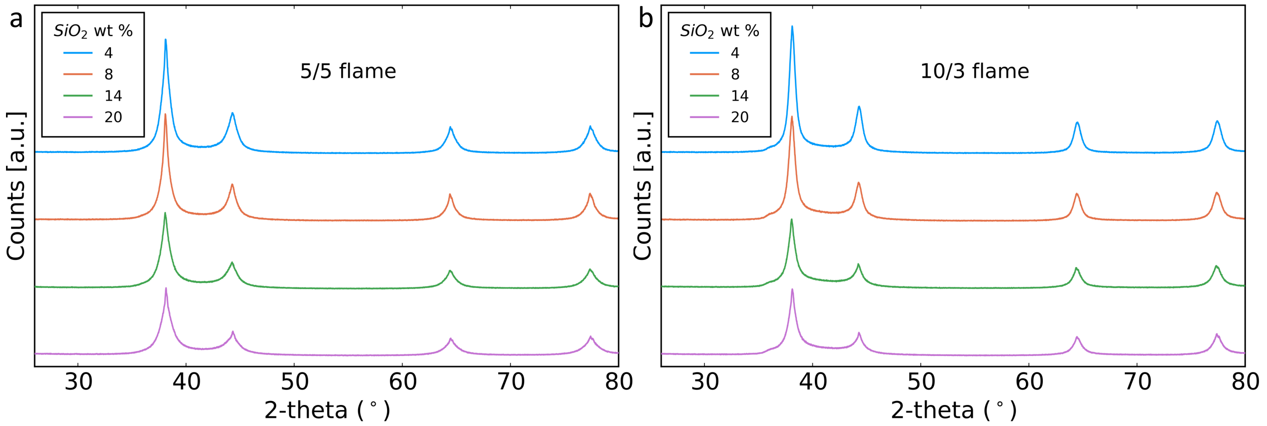

Figure S4: X-ray diffractograms of Ag nanoparticles used for the filter coating experiments at both flame conditions. The terms X/X in the figure refer to the flame parameters of (liquid precursor flow rate [ml/min])/(dispersion oxygen flow rate [L/min]).

Table S1: Specific surface area (SSA) determined by BET theory of AgNPs synthesised with different flame conditions and silica weight percentages. The terms X/X in the table refer to the flame parameters of (liquid precursor flow rate [ml/min])/(dispersion oxygen flow rate [L/min])

SSA m^2/g	4 wt% SiO_2	8 wt % SiO_2	14 wt% SiO_2	20 wt% SiO_2
5/5	59	70	97	118
10/3	43	54	71	86

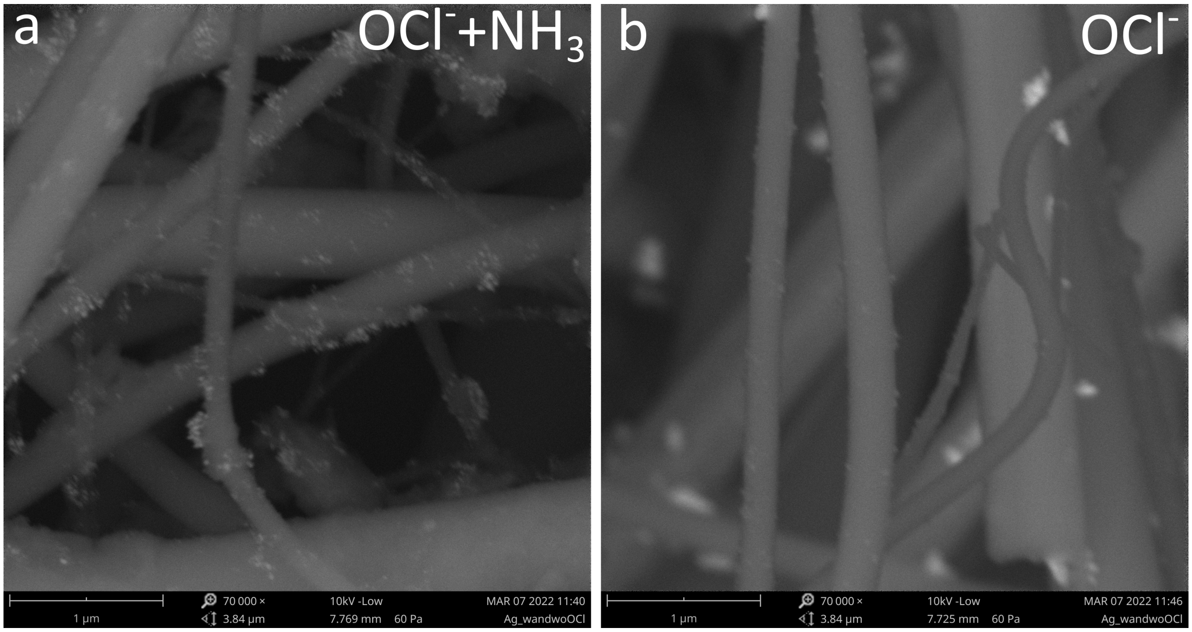

Figure S5: SEM image of the glass fibre filter with AgNPs deposited on it for 5s and treated with OCl^- and 300 µM ammonia (a) and OCl^- alone (b).

4. Materials and methods

Silver nanoparticles were synthesised using flame spray pyrolysis by dissolving silver acetate (99%, Alfa Aesar) in equal volumes of acetonitrile (≥ 99.5%, Alfa Aesar) and 2-ethylhexanoic acid (99%, Sigma Aldrich) under reflux [31] .For the synthesis of silica supported silver nanoparticles (Ag/SiO_2) hexamethyldisiloxane (HMDSO, ≥98 %, Sigma Aldrich) was added to achieve the desired theoretical weight % silica in the synthesised Ag/SiO_2 nanoparticles. Flame spray pyrolysis was performed by using a syringe pump (New Era Pump Systems, Inc.) to flow

the precursor solution at rate of 5 mL/min unless otherwise stated through the burner capillary and subsequently dispersing the liquid stream into fine droplets using pure oxygen at a rate of 5 L/min and a pressure drop of 1.8 bar unless otherwise stated. The fine droplets were ignited by a pre-mixed methane/oxygen flame with flow rates of 1.5 L/min methane (>99.5%, AGA Gas AB) and 3.2 L/min oxygen (>99.5%, Strandmöllen AB). Dry nanoparticle powders of Ag/SiO_2 could be collected by vacuum filtration from the aerosol using glass fibre filters (Hahnemühle, 257 mm, GF6) and a MINK vacuum pump (Busch).

The preparation of SiO_2-coated Ag nanoparticles was performed as described previously,[21,22] in brief the reactor was enclosed with a quartz tube (20 cm height, 4.4 cm wide), pure oxygen was supplied to the flame though a sintered metal ring in the burner at a rate of 40 L/min. HMDSO vapour was prepared from liquid HMDSO at 10°C by bubbling nitrogen through the HMDSO at a rate to achieve the appropriate theoretical silica wt % in the final coated nanoparticles assuming complete HMDSO vapour saturation of the nitrogen gas. The HMDSO vapour laden gas flow was then joined using a V-junction with a nitrogen flow at a fixed flow rate of 21 L/min this was then fed to a metal ring (4.4 cm diameter) with small holes facing into its centre positioned at the top of the quartz tube. A second quartz tube was placed on top of the metal ring. The dry nanoparticle powder was collected as before. Deposition time on the glass fibre filter was controlled by switching on and off the vacuum pump. A minimum distance of 50 cm was maintained between the top of the flame or coating tube and the glass fibre filter.

Transmission electron microscopy was performed by dipping carbon coated copper grids into a dispersion of nanoparticles and drying at room temperature. The grids were then imaged in a Talos 120C G2 with Ceta-D detector and 120 kV LaB6 source. X-ray powder diffractograms were collected from powders using low-background silicon sample holders in a Rigaku Miniflex

equipped with a Cu-K-α source, a Ni filter was used to remove the K-β radiation. The scan rate used was 5 degrees/min. The diffractograms of treated SiO_2-coated Ag nanoparticles were collected from powder by performing treatment for 10 minutes with appropriate conditions in a 10 mL volume of 1 mg/mL dispersed nanoparticles in ultrapure water, washing 3 times with ultrapure water by centrifugation and subsequently freeze-drying to powder.

Absorbance measurements from dispersed nanoparticles were collected using a Spark multi-well plate reader (Tecan) measuring from 300 to 800 nm in 10 nm increments. Ammonia measurements using dispersed nanoparticles were performed in 96 well plates by adding 50 µL of the solution to be measured, 25 µL of NaOCl (0.075 g/L active chlorine @ pH 10.22, prepared with ultrapure water from 30 g/L active chlorine, reagent grade, VWR) and 25 µL of the nanoparticle dispersion (0.5 mg/mL in ultrapure water). Absorbance measurements were immediately started and measured over a 20-minute period. The nanoparticle dispersion was prepared by sonication at 0.5 mg/mL in a cup-horn sonicator (Sonics Vibracell 750) with 60% amplitude for 5 minutes. Separation of ammonia across Nafion membranes was performed using custom 3D-printed cells (see SI for model). The printer used was an Anycubic Photon and the resin was clear PrimaCreator Value UV resin (3Dprima), printer settings used were first 5 layers exposed for 30s, subsequent exposure time 8s, layer height 0.05 mm. The Nafion membrane (N-115 Alfa Aesar) had 8 mm circles punched out which were placed between the two halves of the 3D-printed cells and the cells were clicked together (see Figure 4a for a diagram). The sample side was filled with 550 µL and the collection side was filled with 330 µL of 3M sodium acetate pH 8.4 (pro analysis, Merck). Unless otherwise stated ammonia was allowed to transport across the membrane for 20 minutes, after which time the sodium acetate solution was removed and analysed in a 96 well plate as described earlier.

For ammonia analysis from sheep serum the silver nanoparticle sensor was applied in parallel to the modified Berthelot method. 50 µL aliquots of the Na acetate buffer after 20 minutes were placed in a 96 well plate with 3 aliquots for each detection method. For the silver nanoparticle sensor 30 µL of 0.08 g/L active chlorine NaOCl was added followed by 25 µL of the nanoparticle dispersion (0.5 mg/mL in ultrapure water). For the modified Berthelot method reagents were added in the following order 25 µL of Orthophenyl phenol (2.8 g/L in 0.036 M NaOH), 25 µL of sodium nitroprusside (0.72 g/L in 0.22 M NaOH) and 12.5 µL of Na dinitrocyanide (2.8 g/L in 0.07 M NaOH). The absorbance spectra were recorded as before with a Tecan Spark plate reader.

Measurements directly from glass fibre filter paper were performed by cutting 10 x 1.3 mm strips of filter paper with a 5x10 mm square at one end to increase the volume of liquid drawn up. The nanoparticle coated filter paper was then inserted into a well of a 96 well plate containing 50 µL of sample and 25 µL of NaOCl (0.075 g/L active chlorine) and allowed to stand for 10 minutes after which time it was removed and allowed to dry. Imaging of the glass fibre filter was performed using a Raspberry Pi NOIR module V2 8-megapixel camera attached to a Raspberry Pi Zero processor in a custom 3D printed enclosure with white light LED illumination to ensure consistent illumination and imaging conditions between experiments.
